# Statement from Prof. A. F. A. King, M. D.

**Published:** 1878-03

**Authors:** A. F. A. King

**Affiliations:** 726 13th St., Washington, D. C.


726 13th St., WASHINGTON, D. C.,

February, 7, 1878.

JULIUS F. MINER, M. D.

*Editor "Buffalo Medical and Surgical Journal."*

DEAR SIR :—Will you be kind enough to state in your Journal that I know nothing whatever of "*Cronyn's Boudoir Medicines*," and have never given permission for my name to appear with those who have recommended it. The use of my name in this connection is an usurpation for which I could wish the usurpers were amenable to law.

Very Respectfully Yours,

A. F. A. KING, M. D.

